# Current Challenges and Disparities in the Delivery of Equitable Breast Cancer Care in Canada

**DOI:** 10.3390/curroncol30080527

**Published:** 2023-08-01

**Authors:** Emily B. Jackson, Christine E. Simmons, Stephen K. Chia

**Affiliations:** 1 BC Cancer Vancouver, Vancouver, BC V5Z 4E6, Canada; 2 Department of Medicine, University of British Columbia, Vancouver, BC V6T 1Z3, Canada

**Keywords:** breast cancer, equity, outcomes, barriers to care, populations at risk

## Abstract

Recent exciting advances in the diagnosis and management of breast cancer have improved outcomes for Canadians diagnosed and living with breast cancer. However, the reach of this progress has been uneven; disparities in accessing care across Canada are increasingly being recognized and are at risk of broadening. Members of racial minority groups, economically disadvantaged individuals, or those who live in rural or remote communities have consistently been shown to experience greater challenges in accessing ‘state of the art’ cancer care. The Canadian context also presents unique challenges—vast geography and provincial jurisdiction of the delivery of cancer care and drug funding create significant interprovincial differences in the patient experience. In this commentary, we review the core concepts of health equity, barriers to equitable delivery of breast cancer care, populations at risk, and recommendations for the advancement of health equity in the Canadian cancer system.

## 1. Introduction

Breast cancer represents the most common cancer diagnosis in women, with a one in eight lifetime risk of the diagnosis. It is the second-leading cause of cancer-related deaths in Canadian women [1]. It is estimated that there were over 28,600 breast cancer diagnoses in Canada in 2022 [2]. Fortunately, age-standardized mortality rates from breast cancer have steadily declined over recent decades, owing to many factors including improved treatments and early detection efforts through screening initiatives [1,2]. However, research continues to describe critical disparities in accessing breast cancer care in Canada, leading to an uneven patient experience and survival outcomes nationally.

Previous studies have shown that patients from socially and economically disadvantaged groups, as well as certain racial or ethnic backgrounds, and patients residing in rural settings all experience unique challenges in accessing care, which can in turn impact their disease-specific outcomes [3,4,5,6,7,8,9,10,11]. These problems are undeniably complex, involving the intersection of numerous patient, disease, social, and healthcare system factors, and this remains an understudied field in oncology. We have a responsibility to identify and study these disparities so that we may understand where the greatest gaps in care and survival exist, which factors are potentially modifiable, and which interventions carry the greatest chance for success to ‘level the playing field’.

This paper will define the core concepts of health equity and inequity and then examine key examples in the recent literature that speak to this critical issue. This will provide a conceptual framework for recognizing disadvantaged populations and disparities in clinical care and provide recommendations for the advancement of health equity in the Canadian cancer system.

## 2. Defining Health Equity

Health equity is defined by the World Health Organization as the “absence of unfair, avoidable or remediable differences among groups of people, whether those groups are defined socially, economically, demographically, or geographically or by other dimensions of inequality (e.g., sex, gender, ethnicity, disability or sexual orientation)” [12]. Health equity implies that every person has the opportunity to achieve their full potential for health and well-being and that no one should be prevented from achieving this potential if it can be avoided [13].

The presence of health inequity creates a system where patients from economically and/or socially disadvantaged backgrounds, patients from certain racial or ethnic groups, or other social dimensions experience systemically worse health outcomes than others. In oncology care, practitioners and researchers are appropriately in constant pursuit of new and improved strategies and treatments to advance the status quo and improve cancer outcomes for their patients. However, we must lend equal importance to ensuring that all patients have equitable access to standard, high-quality care and therapies. Just as a chain is only as strong as its weakest link, the caliber and performance of our healthcare system is proportional to its capacity and commitment to deliver care to its most vulnerable populations.

## 3. Understanding How Health Inequity Is Created through an Oncology Lens

Health inequity results from the intersection of many complex, interwoven, and multifaceted issues and can impact a patient at any level of interaction within the healthcare system (e.g., primary, secondary, or tertiary prevention). When categorized into core mechanisms of inequity, there exists at least three key mechanisms:

1. Differential exposure to cancer risk factors and primary prevention techniques.
2. Social stratification creating at-risk populations.
3. Unequal allocation of power and resources leading to differential access to health services.

Furthermore, these mechanisms are not independent from one another but are tightly linked and when experienced together can magnify the resulting disparities. It is crucial that oncology practitioners and researchers understand the innumerable ways that a patient’s medical outcome can be impacted by their social reality, looking beyond just disease-specific factors through to their entire lived experience.

**Figure 1:**
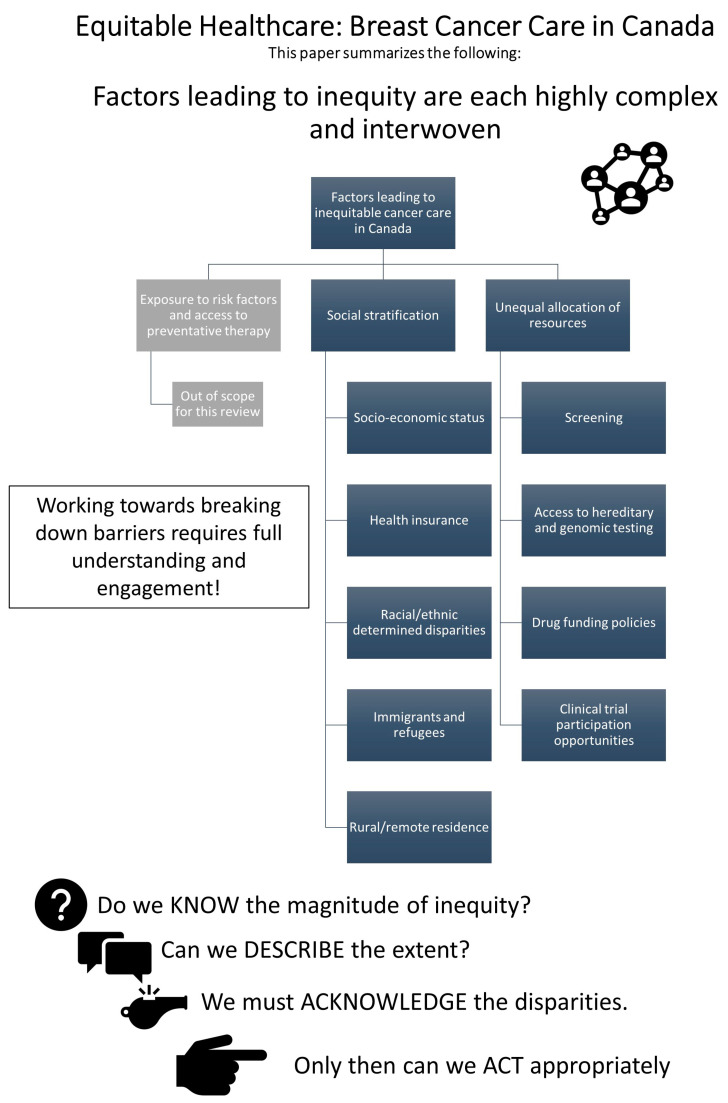
Factors influencing the delivery of equitable breast cancer care in Canada.

In the following subsections, we will focus our discussion on how mechanisms #2 and #3 create inequity in breast cancer care within Canada. Figure 1 offers a graphic representation of the topics addressed in this paper. 

### 3.1. Social Stratification

#### 3.1.1. Socio-Economic Status

Socio-economic status (SES) is a composite measure of a person’s access to financial, educational, social, and health resources. Over three million people live in poverty in Canada (8.1% of the population in 2020), and poverty rates were notably disproportionately higher among some racial groups, non-binary individuals, recent immigrants, and Indigenous peoples, among others [14]. Extensive prior research links lower SES to inferior general health outcomes. Lower income, lower education, and lower employment rates are consistently linked to increased preventable and treatable premature mortality [15].

Lower SES has also specifically been linked to inferior breast cancer outcomes. In a large prospective population-based case-control study of 5820 women aged 20–69 diagnosed with breast cancer and living in Wisconsin, USA, investigators analyzed both individual and community level SES data to study how this impacted breast cancer-specific survival (BCSS) [11]. Individual-level SES data were collected through telephone interview, and community-level SES data were collected through the US census. This was cross-referenced with clinical-pathological and survival data from the state cancer registry. Lower individual and community-level education and income-to-poverty ratios were both associated with lower annual screening mammography use. A lower personal income-to-poverty ratio and community education level were associated with a more advanced stage at diagnosis with breast cancer. This study also found that women with lower education levels (no education beyond high school) and the lowest household income level (<2.5X vs. ≥5X the poverty level) were significantly more likely to die from breast cancer (1.39X [95% confidence interval (CI) 1.10–1.76, *p* < 0.05] and 1.46X [95% CI 1.10–1.92, *p* < 0.05], respectively). After adjusting for several additional factors, including screening mammography use, stage, family history, and lifestyle factors, only community level education was associated with breast cancer mortality [hazard ratio (HR) 1.57 for communities with ≥20% adults without high school degree vs. <10%; 95% CI 1.09–2.27, *p* < 0.05).

Despite its publication over a decade ago, this study remains one of the most robust investigations into the link between SES and breast cancer outcomes to date. While the included population was based exclusively in the USA, comparable studies undertaken in the Canadian context have not yet analyzed community-level SES data with the same granularity and did not include assessment of individual-level SES factors [16].

Nonetheless, findings in the Canadian context were congruent. One Canadian study examined the impact of community-level income determined using patient postal codes and included 34,776 patients diagnosed between 2004–2009 in Ontario [16]. After adjusting for baseline patient demographics, stage at diagnosis, adjuvant chemotherapy and trastuzumab use, adjuvant radiotherapy (RT), and surgery type, higher community-level income was associated with improved overall survival (OS). BCSS analysis was unfortunately not conducted, and thus is a limitation in understanding the impact of breast cancer on attribution of mortality relative to income.

#### 3.1.2. Health Insurance

Access to health insurance can mitigate some of the disparities associated with lower SES. While this can be challenging to address, this effect can be studied by comparing the United States (US) and Canadian contexts due to considerable differences in health insurance coverage between countries. The Canadian oncology healthcare system is delivered by provincial governments and provides universal coverage of physician and nursing-based care in clinic or hospital settings. The specific treatments funded in each province do vary, but all jurisdictions provide radiotherapy and an array of systemic therapy options (see Section 3.2.3. Drug Funding Policies below for more details). In contrast, in the US there is no universal coverage, and individuals rely on private health insurance, Medicare coverage (if over 65 years old, or some people <65 with certain disabilities or conditions), Medicaid (for individuals with limited income or resources), private pay, or a combination thereof. Oncology treatments can be extremely costly, and in particular, the US system can leave many patients with considerable out-of-pocket expenses with standard-of-care therapy [17].

One of the pivotal studies establishing the beneficial effect of private health insurance status on overall survival in patients with breast cancer was conducted in New Jersey, USA, in 1993 [8]. This retrospective population-based cohort study included 4675 women aged 35–64 who had a diagnosis of breast cancer between 1985–1987. Baseline clinical-pathological features and outcomes were compared across cohorts of women with private insurance versus no insurance versus those with Medicaid coverage. Compared to women with private insurance, uninsured and Medicaid-insured women were younger, less likely to be White, less likely to be married, and more likely to live in lower income communities. Uninsured patients and those with Medicaid were significantly more likely to present with advanced disease compared to those with private insurance (rate of de novo metastatic disease 7.3% with private insurance, 12.3% with no insurance (*p* < 0.001), 17.4% with Medicaid coverage (*p* = 0.01)). In a multivariable analysis adjusting for age, race, marital status, median household income, comorbidities, and stage at diagnosis, patients with no insurance or Medicaid coverage experienced a higher risk of death compared to those with private insurance (relative risk (RR) 1.49 (95% CI 1.20–1.84) with no insurance, and RR 1.40 (95% CI 1.04–1.89) with Medicaid coverage). While these results now represent a cohort of patients diagnosed over 30 years ago at a time when treatments differed dramatically from the present day, these results are still relevant and thought provoking as newer treatments are typically even more costly, potentially driving a greater divide in outcomes.

How these results specifically apply to the Canadian context, with universal coverage of physician and hospital-based services, was investigated in a 2009 meta-analysis which included an aggregate population of 130,083 patients diagnosed with breast cancer between 1984–2000 [18]. This study compared low-, middle-, and high-income groups of patients between Canada and the US. Investigators found that lower SES was not strongly associated with BCSS in most of the Canadian contexts studied but was consistently associated with inferior BCSS in the US. In the between-country analysis, there was no difference in survival in the middle- and high-income groups between Canada and the US, but in the low-income patient group, Canadian women were advantaged with an age-adjusted survival rate ratio of 1.14 (95% CI 1.13–1.15, *p* < 0.05). This advantage was even more pronounced in patients <65 years old in the lower income group with an age-adjusted survival rate ratio of 1.21 (95% CI 1.18–1.24, *p* < 0.05). This effect is hypothesized to relate to lack of Medicaid eligibility in US patients under 65 years old. 

The results of this meta-analysis robustly affirm a health insurance theory mitigating the negative effect of lower SES factors and drives home the critical importance that our publicly funded oncology care in Canada should be as inclusive and comprehensive as possible. 

#### 3.1.3. Racially and Ethnically Determined Disparities

The Canadian population is ethnically and culturally diverse. In the 2016 census, over 250 origins were reported, with French and British Isles origins being the most frequent. Over two million people reported Aboriginal ancestry (comprised of First Nations (population 1.5 million, with many subgroups within First Nations), Métis (population 600,000), and Inuit individuals (population 79,125)) [19]. Aboriginal or Indigenous peoples is a collective term referring to these three groups, who are descendants of the original peoples of North America, and each have unique histories, languages, cultures, and spiritual beliefs and practices. Extensive prior research has linked poor general and oncologic health outcomes to certain racial or ethnic identities and patient populations [20,21,22,23,24]. As was critically highlighted in the In Plain Sight report, which revealed serious discrimination in the Canadian and BC healthcare systems in general, Aboriginal patients in Canada are a patient population frequently shown to be at increased risk of poor outcomes [25]. The burden and outcomes of cancer among indigenous peoples in Canada has been understudied for many reasons, one being typically a lack of ethnic identifiers in cancer registries and outcomes databases.

A recently published prospective population-based cohort study of approximately two million respondents to the 1991 Canadian Long Form Census gives poignant evidence of the disparities in oncologic outcomes experienced by First Nations patients [26]. This study linked data from the Long Form Census to the Canadian Cancer Registry, Canadian Mortality Database, and tax summary files. Patients were categorized as First Nation or non-Aboriginal and followed for cancer diagnoses and deaths through the linked registries. First Nations people were more likely to fall within the lowest income quintile, to be younger, and to live in a rural region compared to non-Aboriginal individuals. Excess mortality rate ratios (EMRR) and five-year, age-standardized relative survival rates were calculated for 15 cancers and accounted for age, sex, time period at diagnosis, community-level income quintile, and rurality. Investigators found that First Nations people diagnosed with cancers of the colon, rectum, lung, breast, prostate, oropharynx, cervix, ovary, non-Hodgkin lymphoma, and leukemia all had significantly poorer survival rates than their non-Aboriginal peers. The largest relative differences in survival were observed in breast (EMRR 1.55, 95% CI 1.23–1.95) and prostate cancer (EMRR 1.76, 95% CI 1.14–2.73). These findings are consistent with previous research in this field.

This was the first national study of its kind to date and highlights the stark reality that opportunities for care and well-being are not experienced equally across the nation. Reasons for these disparities are certainly multifactorial, but regardless of the root causes, these findings shed light on critical opportunities for improvement.

#### 3.1.4. Immigrants and Refugees

Immigrant and refugee populations have frequently been shown to be at risk of inferior health outcomes and inequity; indeed, the health status of immigrants has been shown to decline upon arrival in Canada [27].

A population-based study examining the impact of ethnicity on breast cancer stage at diagnosis in Ontario explored this issue. Investigators compared stage at diagnosis with breast cancer between patients of South Asian (n = 705) or of Chinese ethnicity (n = 1304) (identified using a validated surname algorithm) with that of the general population in Ontario (N = 39,287). All patients had a breast cancer diagnosis between 2005–2010. When adjusted for age and compared to the general population, South Asian women were more often diagnosed with breast cancer at stage II than stage I (odds ratio (OR) 1.28, 95% CI 1.08–1.51) or at stage II-IV compared to stage I (OR 1.27, 95% CI 1.08–1.48). Chinese women were less likely to be diagnosed at stage II than stage I (OR 0.82, 95% CI 0.72–0.92) or at stage II-IV than stage I (OR 0.73, 95% CI 0.65–0.82). In this analysis, South Asian and Chinese women were significantly more likely to be under 50 years old at the time of diagnosis compared to the general population, and >60% of the population of both South Asian and Chinese women had been living in Ontario for more than 15 years. Both South Asian and Chinese women had made more visits to their primary care practitioners within the preceding 24 months compared to the general population. South Asian women were also significantly less likely to have undergone prior screening mammography.

These findings are highly relevant to the Canadian context, with a diverse population and large South Asian immigrant group. Ethnic and racial data are rarely collected in provincial cancer registries or outcomes databases, but data deficiency does not mean that inequities in breast cancer care and treatment do not exist. This study reinforces the crucial need to collect these data so that inequities can be accurately studied. 

#### 3.1.5. Geography and Rural Residence

Patients residing in rural and remote locations appear to experience unique challenges in accessing cancer care. Centers offering breast screening and cancer treatments, particularly radiotherapy, tend to be more readily available in urban settings, and this has been shown to impact health resource utilization and breast cancer treatment decisions amongst rural-residing patients.

One retrospective study from British Columbia examined the impact of community size on screening utilization, stage at diagnosis, treatment, and outcomes with breast cancer [10]. Investigators identified all patients with a diagnosis of invasive breast cancer in 2002 (N = 2869) and categorized patients as residing within large, small, and rural local health authorities (LHAs) using Canadian census information. They found that smaller community size was associated with a significantly lower likelihood of screening mammography use (rural vs. urban OR = 0.62, *p* < 0.001) and a higher stage at diagnosis. Urban-residing patients were significantly more likely to pursue lumpectomy and adjuvant radiotherapy, while rural-residing patients were more likely to select mastectomy without radiation. There were no differences in use of chemotherapy or endocrine therapy. Five-year BCSS was 90% for urban-residing patients, 88% for small, and 86% for rural LHAs (*p* = 0.08). Five-year estimates of OS were 84% for urban, 81% for small, and 77% for rural LHAs after a median FUP of 7.4 years (*p* = 0.01). After multivariable analysis controlling for patient, tumor, and treatment factors, the statistical difference in OS did not persist. 

Canada has a uniquely vast geography with approximately 40% of the Canadian population living outside of an urban center, further amplifying the importance of understanding the additional challenges faced by these individuals [28].

### 3.2. Unqeual Allocation of Resources

#### 3.2.1. Cancer Screening

Screening mammography is the most effective and well-established practice to promote early detection and treatment of breast cancer, and publicly funded programs exist in all provinces. Utilization of screening resources has generally increased in Canada over recent decades, but the degree of participation still varies significantly across certain patient populations. A 2015 population-based study in urban Ontario showed disparities in breast cancer screening by immigration status [29]. Researchers linked social and health databases in Ontario to determine the proportion of women who were screened between 2010–2012 among the total population of approximately 1.4 million screen-eligible women residing in an urban setting within Ontario. Included women were grouped into three separate and mutually exclusive categories according to their immigration status: identified immigrants (n = 183,332, 13%), recent registrants with the provincial health plan (n = 63,678, 5%), and long-term residents (Canadian-born or immigrated prior to 1985) (n = 1,160,050, 82%). The identified immigrant group was further subdivided by length of time in Canada into new immigrants (≤5 years), recent immigrants (6–10 years), and established immigrants (11 years or more).

There were some important differences in baseline demographic factors between the groups, with identified immigrants having the highest proportion of women residing in the lowest two neighborhood income quintiles and being more likely to undergo periodic health exams and visits to their family physician. Investigators found that within the total screen-eligible population, 64% of women underwent mammography. Screening rates by subgroup revealed 57% of identified immigrants, 57% of recent registrants, and 66% of long-term residents underwent screening. After multivariable analysis, immigrants had significantly lower rates of screening compared to long-term residents with an adjusted rate ratio (ARR) of 0.87 [95% CI 0.85–0.88] for new immigrants, ARR 0.90 [95% CI 0.89–0.91] for recent immigrants, and ARR 0.96 [95% CI 0.96–0.97] for established immigrants.

This study shows persistent suboptimal screening practices among urban immigrants to Canada and highlights just one of many important ways that various patient populations experience a unique version of healthcare across our system.

#### 3.2.2. Availability of Hereditary and Genomic Testing

Hereditary and genomic sequencing has frequently been shown to be unevenly applied across patient populations, with patient race being a key influence. A 2017 retrospective population-based cohort study in Florida, USA, revealed critical disparities in access to hereditary testing in women with breast cancer diagnosed at age ≤ 50 and also uptake of risk-reducing therapies in those ultimately diagnosed with a pathogenic BRCA mutation [9]. Included patients (N = 1622) were subdivided by self-reported ethnicity into four subgroups: Black (n = 440), Spanish-speaking Hispanic (n = 117), English-speaking Hispanic (n = 168), and non-Hispanic White (NHW) (n = 897). Researchers found that Black patients were significantly less likely to have discussed genetic testing with their healthcare provider compared to NHW patients (37% vs. 86%, OR 0.06, 95% CI 0.04–0.10, *p* < 0.0001). They were also significantly less likely to have completed genetic testing compared to NHW patients (36% vs. 65%, OR 0.18, 95% CI 0.11–0.29, *p* < 0.0001). Spanish-speaking Hispanic patients were also less likely to have discussed genetic testing compared to NHW patients (70% vs. 86%, OR 0.54, 95% CI 0.30–0.96, *p* = 0.04), but there were no differences observed in completion of genetic testing between these groups. There were also no differences observed in the rates of discussion and testing between English-speaking Hispanic and NHW patients.

In a multivariable analysis, after controlling for socioeconomic, demographic, and disease-specific factors, Black patients were less likely to have discussed genetic testing with their physician and less likely to have completed genetic testing compared to NHW patients. The differences between Spanish-speaking Hispanic and NHW patients were no longer significant. 

Among the subpopulation of patients who were later identified to have a pathogenic BRCA mutation (n = 90), there were also significant differences in the uptake of risk-reducing salpingo-oophrectomy (RRSO) and risk-reducing mastectomy (RRM) by subgroup. RRSO rates were 28.1% for Black patients, 76.6% for NHW patients, and 90.9% for Hispanic patients (both English- and Spanish-speaking). RRM rates were 68.8% for Black patients, 95.7% for NHW, and 81.8% for Hispanic patients. In a multivariable model controlling for confounders, with NHW and all Hispanic patients together as the reference group, Black patients remained significantly less likely to undergo RRSO (*p*= 0.025) and RRM (*p* = 0.008).

This study remains one of the sole population-based efforts to compare differences in both hereditary testing and subsequent downstream cancer management practices across racial and ethnic minority populations. These findings draw attention to a very concerning health system access disparity in the USA and raises the question of how the results of a similarly designed study in Canada would compare. How these observed differences will continue to impact patient opportunities for treatment and risk reduction in the future with newer and costly treatments such as adjuvant PARP-inhibitor therapy is unclear. However, unfortunately, gaps in care do not typically diminish with the introduction of more expensive options, and so disparities may persist or even widen with further missed opportunities.

#### 3.2.3. Drug Funding Policies

Following approval by Health Canada, all oncology medications undergo assessment by the Canadian Agency for Drugs and Technologies in Health (CADTH) pan-Canadian oncology drug review (pCODR). pCODR reviews the clinical evidence, economic impact, and patient-important aspects of cancer drugs that have been approved by Health Canada. Next, pCODR makes funding recommendations to the provinces (with the exception of Québec, who does not participate in pCODR). Québec has its own provincial organization and review body called the Institut national d’excellence en santé et en services sociaux (INESSS). The provincial agencies (all but Québec) then must make a final decision regarding drug coverage, taking into consideration CADTH recommendations and other provincial economic realities. Québec makes funding decisions after a similar review process of each drug by INESSS.

While the Canadian population is generally fortunate to have publicly funded access to most established standard-of-care therapies for cancer, there are several challenges and limitations. Funding decisions can be inconsistent between provinces, and there can be significant lags between the phases of Health Canada approval, pCODR assessment, and then an ultimate positive funding decision and listing by each individual province. For example, there was more than a 17-month lag in a positive funding decision for pertuzumab for HER2-positive advanced breast cancer between the first province to offer funding (British Columbia) and the last (Prince Edward Island) [30]. The existence and impact of these lags in funding have been studied in detail and arguably risks creating a dramatically different patient care experience depending on provincial residency [31,32,33,34]. Previous research by Gotfrit et al. also shows substantial potential total life-years lost (39,067 life-years lost between 2011–2016) experienced by patients due to the lag in time between proof of efficacy until a positive funding decision is made [34]. It is likely that patients residing in provinces typically slower to implement funding would experience a disproportionate burden of life-years lost.

Funding of take-home oral cancer medications is also fragmented—some provinces (e.g., Ontario) do not provide a comprehensive funding plan for these mediations and access must be obtained through other means such as Patient Access Programs offered by pharmaceutical companies, private health insurance, or specialty drug coverage programs offered through Provincial governments (e.g., Ontario Drug Benefits or Trillium Drug Program). This system is unnecessarily complex, can be labor intensive, and risks leaving some groups of patients with suboptimal coverage, potentially delays in treatment initiation, and costly out-of-pocket co-payments from patients.

Until there exists a more uniform method of funding for all cancer medications across Canada, inequity in drug access is inevitable. How this specifically affects outcomes in our population with breast cancer requires further study.

#### 3.2.4. Clinical Trial Participation Opportunities 

Clinical trials are essential to advancing oncology care and outcomes, offer a rigorous support network for participants, and can provide novel therapeutic options. Despite a mandate to support equity, diversity, and inclusion initiatives in clinical research and to recruit representative patient populations to clinical trials, certain patient populations remain underrepresented. Many possible factors can influence opportunities for enrollment, including geography, race and ethnicity, socio-economic status, age and comorbidities, and gender, among others.

A recent study conducted in the United States found disproportionately low participation of racial and ethnic minority populations in phase one drug trials for metastatic cancer [35]. This cross-sectional study examined the proportions of racial and ethnic groups represented in all phase one trials where race was reported (N = 221) and conducted in the US between 2000 and 2018, and then compared enrollment proportion with the true incidence of each race and ethnicity with metastatic cancer within the total US population as determined by a central database. Approximately 26% of the examined clinical trials focused on metastatic breast cancer. Investigators found a relative over-representation of White patients and under-representation of Asian, Pacific Islander, and Black patient participants. When patient participation was compared longitudinally by study time period (2000–2011 vs. 2012–2018), there was an increase in the over-representation of White patients and a corresponding decrease in representation over time of American Indian or Alaskan Native, Asian or Pacific Islander, Black patients, and Hispanic patients. Disproportionate participation in trials is multifactorial and highly complex and can result from systemic biases or lack of inclusive initiatives, elements of mistrust due to the history of harms experienced by some racial groups at the hands of the healthcare system, geography and patient drive distance to the clinical research center, language barriers, and cultural beliefs, among other factors.

Similar studies conducted in Canada are generally consistent, showing that certain patient populations are under-represented and total enrollment on a population scale is lower than other similar countries [36]. Ultimately, this widening gap in representation shown by Dunlop et al. is deeply concerning [35]. Oncologists and researchers must interpret this data as an urgent call to action to address these growing inequalities.

## 4. Moving Forward

Disparities in access to healthcare result from the intersection of inherently complex and interwoven factors, and often these influences begin long before a person even makes contact with the healthcare system and becomes a patient. Healthcare practitioners, researchers, and allied health professionals have a responsibility to seek to understand these complex factors at the patient, system, and societal level. Understanding where disparities stem from and describing and quantifying gaps in healthcare is just the first step in creating a more equitable healthcare system in Canada.

There is a paucity of local data. Most of the studies discussed herein refer to the US context, chosen for discussion mainly because there are few studies conducted in Canada. There is an urgent need for Canadian clinicians and investigators to document the nature and severity of the sequelae from inequity in oncology care. Institutions and educational grants are increasingly in support of research in this field, but there is considerable work remaining and this will take time. In order to support this research, large population-based databases must capture additional demographic factors such as sex, gender, self-reported ethnicity, private health insurance coverage, household income, and other SES factors wherever possible and feasible.

Once gaps in care are described, targeted action strategies can be planned and implemented to mitigate the disparities experienced by the identified vulnerable populations (Table 1). The Canadian oncology community must encourage enrollment of diverse patient populations into clinical trials and fiercely advocate for uniform access to new systemic therapies for all eligible patients. There are many successful ongoing examples of such initiatives. For example, the CRAFT framework initiative aimed to increase clinical trial opportunities in rural and remote locations in Canada [36]. In addition, many cancer centers across the country have a dedicated Indigenous Cancer Patient Navigator program and personnel to help this patient population through interactions with the healthcare system and as they progress through their journey with their cancer, all while respecting their cultural ecosystem. Mobile mammography units also provide a form of screening outreach in many provinces, helping to mitigate some of the unique challenges that patients in rural and remote communities face.

Inherent in this discussion and the success of any initiatives that would follow is recognizing the fundamental importance of cultural competency and a commitment to person-centered care. The design and implementation of any strategy should involve key stakeholders, most importantly including the relevant patient group(s) themselves. Weaving these core values through our healthcare system begins at the individual practitioner level and should begin early in medical education. This is essential to meet the unique physical, psychosocial, and cultural needs of every patient.

## 5. Conclusions

Oncology care is becoming increasingly complex and costly, which fortunately has led to much-needed improvements in quality of life and survival for patients living with breast cancer. However, not all patients experience these advancements to the same degree. Disparities in accessing high-quality cancer care exist across Canada, and it is collectively our responsibility to understand the root causes of these inequities so that we can advance the standard of care for all individuals living with cancer across Canada. 

## Figures and Tables

**Table 1:** Suggested areas of focus and intervention to reduce disparities in breast cancer care in Canada.

Factor	Action Plan
What impacts a patient’s risk and cancer care experience?	To understand the diverse factors that influence risk of cancer and their outcomes, we must broaden the characteristics captured in large population-based databases wherever possible. Can we include gender, sex, ethnicity, health insurance, household income, and other socio-economic factors?
Identify groups at risk and quantify gaps.	Use population-based databases to identify populations who experience worse cancer outcomes. We must generate local Canadian data.
How can we better support vulnerable populations as they access care?	How can at-risk populations be better supported as they migrate through the cancer care system? Targeted analysis of interventions should be involved, such as dedicated nurse navigators, mammography outreach programs, community-based infusion clinics closer to home, among other interventions identified through research and discussion with key stakeholders, including patient stakeholders.
How can we better integrate all patients into clinical research opportunities?	Continue researching what patient populations have more difficulty accessing clinical trial participation opportunities and why these challenges exist. Design targeted interventions to promote inclusive and broad enrolment. How do we promote enrollment from rural areas? From ethnically diverse groups? From patients of all socioeconomic backgrounds?
Establish and strengthen partnerships.	Develop strong connections with patients, public, private, and volunteer programs, and organizations as a critical commitment to person-centered care. Weave cultural competency into our healthcare system starting from early medical education.

## Data Availability

No new data were created or analyzed in this study. Data sharing is not applicable to this article.
